# THE PMDC AS A FRAMEWORK FOR IDENTIFYING AND ORGANIZING PERSON-CENTERED CARE INTERVENTIONS: AN INTEGRATIVE REVIEW

**DOI:** 10.1093/geroni/igae098.1097

**Published:** 2024-12-31

**Authors:** Bill Buron, Tim Killian

**Affiliations:** The University of Tulsa, Tulsa, Oklahoma, United States; University of Arkansas, Fayetteville, Arkansas, United States

## Abstract

Residents living with dementia (RLWD) in nursing homes (NHs) have cognitive and communication deficits. Person-centered care (PCC) practices ranging from improving bathing experiences to supporting communication skills have been the mainstay of non-pharmacologic dementia care treatment, although there are considerable variations in how PCC practices are conceptualized and implemented. In efforts to meet Kitwood’s (1997) challenge to promote personhood among RLWD, Buron (2008) developed the Personhood Model for Dementia Care (PMDC) to identify and categorize three levels of personhood: biologic, individual, and sociologic. Through this integrative review, the researchers use Buron’s PMDC as a framework to intuitively identify, organize and analyze recent literature on PCC interventions. The study leveraged new Artificial Intelligence (AI) tools to identify relevant studies published in the past 5 years (2019-present). The researchers used AI tools to identify over 100 qualifying studies. Through a second phase manual review of the abstracts, the researchers applied the inclusion criteria; reducing the number of papers to approximately 35. Most interventions focused on individual personhood, followed respectively by sociologic and biologic interventions, although there was overlap, with some papers addressing more than one dimension of personhood as defined by the PMDC. In the third phase, the researchers focused on downloading PDF versions of these studies and utilizing Natural Language Processing (NLP) tools to characterize and describe the current landscape of PCC interventions in NHs, using the PMDC as a model to promote additional research and advance the provision of targeted PCC interventions for RLWD in NH settings.

